# Subcellular metabolomics: the choice of method depends on the aim of the study

**DOI:** 10.1093/jxb/erx406

**Published:** 2017-11-16

**Authors:** Karl-Josef Dietz

**Affiliations:** Department of Biochemistry and Physiology of Plants, Faculty of Biology, Bielefeld University, Universitätsstr. Germany

**Keywords:** Cellular compartments, chloroplast, compartmentation, metabolite profiling, non-aqueous fractionation, storage, subcellular metabolomics

**Plant metabolism is compartmentalized at the level of organs, cells, subcellular compartments and molecular assemblies. The rapid progress of in-depth metabolite profiling and kinetic flux analysis has elucidated the environmentally dependent dynamics of metabolism. However, for a full understanding of metabolism and compartmentation, including modelling and simulation (e.g. of photosynthesis), metabolome analysis must consider subcellular localization. Here, the advantages and drawbacks of methods developed to analyze this are assessed. It is argued that the selection of method depends on the aim of a particular study, and results (using different methods) should ideally be compared: no single method is best.**

The methods that have been developed to scrutinize subcellular compartmentation for metabolome analysis include aqueous isolation of organelles, non-aqueous isolation of chloroplasts and non-aqueous fractionation of tissues.

## Aqueous fractionation

Aqueous fractionation necessarily processes tissue in an unquenched metabolic state. Two major potential drawbacks for metabolome analysis following this process need to be considered: the continuation of metabolism and the loss of metabolites to the suspension medium. Metabolites may be lost during irreversible or reversible permeabilization of the organelles and by physiological transport mechanisms present in the boundary membranes. Thus the experimentalist takes advantage of (i) low temperature, usually close to 0 °C, to slow down metabolism and transport; and (ii) fast processing to minimize the time elapsed between mechanical homogenization or maceration of the tissue and isolation of the final fraction for analysis.

Aqueous isolation of intact chloroplasts with subsequent sedimentation and freezing can be achieved in less than a minute (Schröppel-Meier and Kaiser, 1988). However, turnover times of intermediates such as those of the Calvin–Benson cycle are in the range of seconds (Szecowka *et al.*, 2013), and calculated turnover times of ATP and NADPH are significantly below a second. Thus metabolism-dependent alterations and metabolite loss will occur during preparation. Aqueous isolation of other organelles such as mitochondria and vacuoles usually requires even longer time periods. Rapid fractionation of protoplasts by lysis and passage through filters of different cut-offs is an interesting and quick method of assessing subcellular metabolite levels (Gardestrom and Wigge, 1988). However, protoplasts isolated in this way behave differently from cells in intact tissue, with drastically changed metabolite levels and altered metabolism, as the process usually involves enzymatic maceration over hours.

## Non-aqueous fractionation

For non-aqueous fractionation, metabolism is rapidly arrested by deep freezing in liquid nitrogen (Behrens and Thalaker, 1957; Heber, 1957). Freeze drying at less than –40 °C maintains the metabolic state and metabolite distribution of the tissue *in vivo*. Homogenization in organic solvents such as petrolether or hexane combined with tetrachloromethane (or nowadays the less-toxic tetrachlorethylene), followed by fractionation in density gradients made from these organic compounds at proper mixing ratios, allows the isolation of a chloroplast fraction which can further be freed from lighter and heavier cellular material by sedimentation centrifugation. The non-aqueous chloroplast fraction obtained is highly enriched and a very good proxy for the metabolite contents of the chloroplast *in vivo*. The accuracy can be further increased by introducing corrections for contamination based on marker enzymes. The stability of the selected marker enzymes during lyophilization and fractionation should be tested, as any differential loss could affect the final results. Thus up to 80% of NADP-GAPDH activity, a commonly used chloroplastic marker, can be lost during lyophilization and a further 60% during fractionation. The loss of hydrophilic metabolites is extremely low and even lipophilic metabolites leak out slowly, probably due to their incorporation into precipitated, denatured protein particles. Non-aqueous chloroplasts have also been used to dissect dynamic processes of metabolites of low abundance and high turnover (Heber and Santarius, 1965).

The method of non-aqueous isolation of chloroplasts was subsequently advanced to the level of whole-tissue fractionation. Following freeze-drying, the plant tissue is homogenized and subjected to continuous density-gradient centrifugation using hexane and tetrachlorethylene. Several fractions are collected from the gradient and analyzed for both the distribution of specific subcellular markers and the distribution of the metabolite(s) of interest. This approach was first developed by Gerhardt and Heldt (1984), who collected six fractions. The subcellular distribution of metabolites is calculated on the bases of assumed fixed ratios of compartment-specific metabolite levels and compartment-specific markers. Commonly used markers are α-mannosidase for the vacuole, phosphoenolpyruvate carboxylase for the cytoplasm and NADPH-dependent glyceraldehyde-3-phosphate dehydrogenase for the chloroplast. The subcellular metabolite levels are estimated using an iterative calculation.

With the advent of metabolite profiling by GC-MS and LC-MS, non-aqueous fractionation has received revived attention (Arrivault *et al.*, 2014; Krueger *et al.*, 2014; Fürtauer *et al.*, 2016; Hoermiller *et al.*, 2017). The non-aqueous isolation of highly purified chloroplasts developed by Ulrich Heber remains a valuable approach for determining chloroplast metabolite levels *ex vivo* and characterizing the chloroplast metabolome as it varies with different physiological conditions (Heber *et al.*, 1986). Another advantage of this method is that it can easily be implemented in any laboratory. An example of this kind of approach is presented by Hossain *et al.* (2017), investigating the subcellular distribution of various metabolites in sugar beet exposed to high salt concentrations.

## Criteria and problems to consider

When interpreting the results from gradient fractionation and selecting a proper method for subcellular metabolomics, six criteria and problems need to be discussed (Table 1).

**Table 1.** Comparison of methods for assessing subcellular metabolomics

Parameter	Aqueous organelles	Non-aqueous chloroplasts	Non-aqueous fractionation
Purity of fractions	(Often) very high	High	Low
Leakage	Leakage of compounds with active transport and breakage	No leakage of hydrophilic compounds, low leakage of lipophilic compounds	No leakage of hydrophilic compounds, low leakage of lipophilic compounds
Continuation of metabolism	Yes	No	No
Contamination correction	Not needed	Preferred	Fundamental to method
Accuracy	High for stable compounds, low for metabolites with high turnover and efflux transporters	High for compounds absent from the vacuole	Iterative solution, approximation
Advantages	Proper determination of stable compounds	Proper estimate of chloroplast levels	Estimates metabolite levels in various compartments
Disadvantages	Instability of organelles, duration of preparation	Little definition of extra-chloroplast levels	Simplified assumption of uniform distribution of marker enzymes

### (1) Cellular heterogeneity of plant tissues

A plant tissue consists of different, often specialized, cells such as the mesophyll, epidermis, guard cells and several cell types of the vascular bundle (Box 1). These cell types have distinct metabolic programs, accumulate unique metabolite profiles and participate in whole-tissue metabolite exchange at different rates. Thus barley leaves accumulate 5–15% of the leaf glucose and fructose pool in the epidermis during the day, while sucrose remains absent from the epidermis (Dietz *et al.*, 1994). Likewise certain epidermal amino acid pools such as Gly and Phe reach more than 20% of the total leaf pool, while the Arg, Gln and Lys pools represent as little as 1, 6 and 8% of the leaf content, respectively.

**Box 1. Schematic leaf and cell cross sections and shares of compartments to total leaf and cell volumes**

(A) Cross section showing the distribution of different cells in a typical Arabidopsis leaf (C_3_ photosynthesis). (B) Subcellular compartments in a barley mesophyll cell. (C) Relative cellular volumes of different cell types in barley and spinach leaves (above) and compartments/organelles in their leaf mesophyll cells (below). The schematic is based on the electron micrograph shown in Winter *et al.* (1993); data were taken from Winter *et al.* (1993, 1994). Guard cells, cell walls, peroxisomes, Golgi apparatus and endoplasmatic reticulum (ER) were not resolved in these studies.

**Figure:**
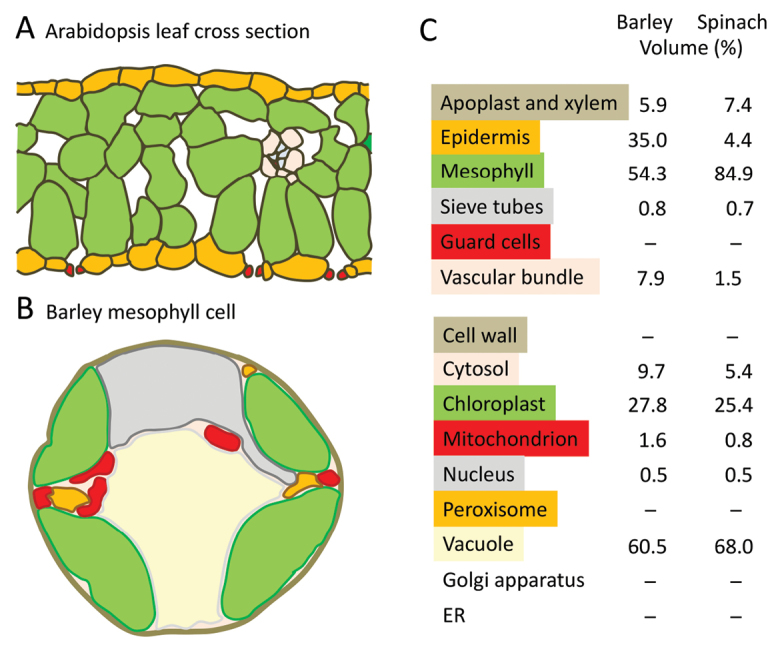

Such differences in concentrations and dynamics prevent any assumption of cellular uniformity when calculating subcellular metabolite levels: a constant ratio of compartment-specific metabolite level and marker level is an oversimplification. Metabolite-to-marker ratios often differ between the mesophyll, epidermis, phloem parenchyma cell and guard cell.

### (2) Missing cellular compartments

Several compartments have so far been omitted from the analysis of subcellular metabolomics, in particular the peroxisomes, cell wall, endomembrane compartments, and also often mitochondria (Box 1). However, these represent significant volumes in the cells and also accumulate specific metabolite profiles. The omission is necessary with the methodology currently available, but weakens the accuracy of iterative approximation of metabolite distribution. It would be extremely valuable to develop methods which include peroxisomes in such analyses, such as in the context of assessing metabolite exchange during photorespiration (Obata *et al.*, 2016).

### (3) The lack of reliable markers

The commonly used vacuolar markers are not exclusively localized to the vacuole. Thus α-mannosidase, like other hydrolases, is found in the vacuoles of mesophyll cells and any other vacuolated cell type such as the epidermis, but in addition is associated with the cell wall and endomembrane compartments (Von Schaewen *et al.*, 1993; Brune *et al.*, 1994). The α-mannosidase activity on a protein basis is >7-fold higher in epidermal cells than in the mesophyll (Dietz *et al.*, 1992; 1994). Thus there exist several compartment-specific ratios of metabolite to marker (e.g. sucrose to α-mannosidase). Recently, other vacuolar markers have been used such as soluble acid invertase and nitrate (Krueger *et al.*, 2014), but the drawback of non-uniform distribution remains. Despite this, calculation of subcellular metabolite distribution from gradient fractions is based on a single value for this decisive parameter.

### (4) Uneven leakiness and metabolite turnover

Metabolite efflux to the medium occurs even during rapid isolation of aqueous organelles. Therefore rapidly isolated aqueous chloroplasts only appear suitable for assessing element and ion distribution and for quantifying abundant metabolites with long turnover times, such as sugars (Schröppel-Meier and Kaiser, 1988). Efflux of any specific metabolite depends on the presence and type of transporters in the boundary membrane and the degree of transient or permanent leakiness or breakage of the organelles during isolation. Levels of metabolites with low abundance and significant turnover cannot be estimated from aqueously isolated organelles.

### (5) Condition-dependent and circadian dynamics

The accumulation, distribution and turnover of metabolites change with developmental state, environmental conditions and circadian status. Rapid changes in metabolite levels, for example in mesophyll cells, will affect the level of these metabolites in other cells with non-uniform kinetics. Such kinetic uncoupling aggravates the estimation of subcellular metabolite concentrations from non-aqueous gradient fractions because the assumed constant metabolite-to-marker ratio is, in reality, a function of time and space (as additional variables).

### (6) Lack of an exact mathematical solution

An iterative search for the best solution of the metabolite-to-marker distribution in subcellular compartments is a reasonable approach when using the fractionation method. However, it should be kept in mind that this will only provide an approximation of the real distribution in the light of the many variables and the limited number of analytical fractions. An exact mathematical solution is impossible.

## Conclusions

From these considerations it is concluded that there is no single and exclusively valid method for subcellular metabolomics. It is necessary to compare the results obtained with different and competing methods to allow dispassionate scrutiny.

The choice of method must depend on the aims of any particular study. Naturally, this choice also depends on the feasibility and availability of resources in a particular laboratory. The isolation of highly purified non-aqueous chloroplasts can be established in most biochemical labs within short periods of time, and allows a good approximation of metabolite changes in the chloroplast. By contrast the analysis and evaluation of continuous non-aqueous gradient fractions requires extensive optimization and considerable resource investment. The advantage is that this method approximates the *in vivo* metabolite state of additional cellular compartments. However, the reliability of data must be considered in the light of the critical points discussed.

## References

[CIT0001] Arrivault S, Guenther M, Florian A 2014 Dissecting the subcellular compartmentation of proteins and metabolites in arabidopsis leaves using non-aqueous fractionation. Molecular & Cellular Proteomics 13, 2246–2259. 24866124 10.1074/mcp.M114.038190 PMC4159647

[CIT0002] Behrens M, Thalaker R 1957 Gewinnung von Chloroplasten in nichtwässrigem Milieu. Die Naturwissenschaften 44, 621.

[CIT0003] Brune A, Urbach W, Dietz KJ 1994 Zinc stress induces changes in apoplasmic protein content and polypeptide composition of barley primary leaves. Journal of Experimental Botany 45, 1189–1196.

[CIT0004] Dietz KJ, Hollenbach B, Hellwege E 1994 The epidermis of barley leaves is a dynamic intermediary storage compartment of carbohydrates, amino acids and nitrate. Physiologia Plantarum 92, 31–36.

[CIT0005] Dietz KJ, Schramm M, Betz M, Busch H, Zink C, Martinoia E 1992 Characterization of the epidermis of barley primary leaves. 1. Isolation of epidermal protoplasts. Planta 187, 425–430. 24178135 10.1007/BF00199959

[CIT0006] Fürtauer L, Weckwerth W, Nägele T 2016 A benchtop fractionation procedure for subcellular analysis of the plant metabolome. Frontiers in Plant Science 7, 1912. 28066469 10.3389/fpls.2016.01912 PMC5177628

[CIT0007] Gardeström P, Wigge B 1988 Influence of photorespiration on ATP/ADP ratios in the chloroplasts, mitochondria, and cytosol, studied by rapid fractionation of barley (*Hordeum vulgare*) Protoplasts. Plant Physiology 88, 69–76. 16666282 10.1104/pp.88.1.69 PMC1055527

[CIT0008] Gerhardt R, Heldt HW 1984 Measurement of subcellular metabolite levels in leaves by fractionation of freeze-stopped material in nonaqueous media. Plant Physiology 75, 542–547. 16663663 10.1104/pp.75.3.542 PMC1066952

[CIT0009] Heber U 1957 Über die Lokalisation von löslichen Zuckern in der Pflanzenzelle. Berichte der Deutschen Botanischen Gesellschaft 70, 371–382.

[CIT0010] Heber U, Neimanis S, Dietz KJ, Viil J 1986 Assimilatory power as a driving force in photosynthesis. Biochimica et Biophysica Acta 852, 144–155.

[CIT0011] Heber UW, Santarius KA 1965 Compartmentation and reduction of pyridine nucleotides in relation to photosynthesis. Biochimica et Biophysica Acta 109, 390–408. 4379647 10.1016/0926-6585(65)90166-4

[CIT0012] Hoermiller II, Naegele T, Augustin H, Stutz S, Weckwerth W, Heyer AG 2017 Subcellular reprogramming of metabolism during cold acclimation in Arabidopsis thaliana. Plant, Cell & Environment 40, 602–610. 10.1111/pce.12836 27642699

[CIT0013] Hossain MS, Persicke M, ElSayed AI, Kalinowski J, Dietz KJ 2017 Metabolite profiling at the cellular and subcellular level reveals metabolites associated with salinity tolerance in sugarbeet. Journal of Experimental Botany 68, 5961–5976. 29140437 10.1093/jxb/erx388 PMC5854137

[CIT0014] Krueger S, Steinhauser D, Lisec J, Giavalisco P 2014 Analysis of subcellular metabolite distributions within *Arabidopsis thaliana* leaf tissue: a primer for subcellular metabolomics. Methods in Molecular Biology 1062, 575–596. 24057387 10.1007/978-1-62703-580-4_30

[CIT0015] Obata T, Florian A, Timm S, Bauwe H, Fernie AR 2016 On the metabolic interactions of (photo)respiration. Journal of Experimental Botany 67, 3003–3014. 27029352 10.1093/jxb/erw128

[CIT0016] Schröppel-Meier G, Kaiser WM 1988 Ion homeostasis in chloroplasts under salinity and mineral deficiency. Plant Physiology 87, 828–832. 16666233 10.1104/pp.87.4.828 PMC1054854

[CIT0017] Szecowka M, Heise R, Tohge T 2013 Metabolic fluxes in an illuminated Arabidopsis rosette. The Plant Cell 25, 694–714. 23444331 10.1105/tpc.112.106989 PMC3608787

[CIT0018] von Schaewen A, Sturm A, O’Neill J, Chrispeels MJ 1993 Isolation of a mutant Arabidopsis plant that lacks N-acetyl glucosaminyl transferase I and is unable to synthesize Golgi-modified complex N-linked glycans. Plant Physiology 102, 1109–1118. 8278542 10.1104/pp.102.4.1109 PMC158895

[CIT0019] Winter H, Robinson DG, Heldt HW 1993 Subcellular volumes and metabolite concentrations in barley leaves. Planta 191, 180–190.

[CIT0020] Winter H, Robinson DG, Heldt HW 1994 Subcellular volumes and metabolite concentrations in spinach leaves. Planta 193, 530–535.

